# Complexes of Ibuprofen Thiazolidin-4-One Derivatives with β-Cyclodextrin: Characterization and In Vivo Release Profile and Biological Evaluation

**DOI:** 10.3390/pharmaceutics15102492

**Published:** 2023-10-19

**Authors:** Ioana Mirela Vasincu, Maria Apotrosoaei, Florentina Lupascu, Andreea-Teodora Iacob, Simona-Eliza Giusca, Irina-Draga Caruntu, Narcisa-Laura Marangoci, Anca Roxana Petrovici, Gabriela Dumitrita Stanciu, Bogdan-Ionel Tamba, Bianca-Stefania Profire, Alin-Viorel Focsa, Mariana Pinteala, Lenuta Profire

**Affiliations:** 1 Department of Pharmaceutical Chemistry, Faculty of Pharmacy, “Grigore T. Popa” University of Medicine and Pharmacy from Iasi, 16 University Street, 700115 Iasi, Romania; ioana-mirela.vasincu@umfiasi.ro (I.M.V.); apotrosoaei.maria@umfiasi.ro (M.A.); florentina-geanina.lupascu@umfiasi.ro (F.L.); andreea.panzariu@umfiasi.ro (A.-T.I.); 2 Department of Morphofunctional Sciences, Faculty of Medicine, “Grigore T. Popa” University of Medicine and Pharmacy from Iasi, 16 University Street, 700115 Iasi, Romania; irina.caruntu@umfiasi.ro; 3 Centre of Advanced Research in Bionanoconjugates and Biopolymers, “Petru Poni“ Institute of Macromolecular Chemistry, 41A Grigore Ghica Voda Alley, 700487 Iasi, Romania; nmarangoci@icmpp.ro (N.-L.M.); petrovici.anca@icmpp.ro (A.R.P.); pinteala@icmpp.ro (M.P.); 4 Advanced Research and Development Center for Experimental Medicine (CEMEX) “Prof. Ostin C. Mungiu”, “Grigore T. Popa” University of Medicine and Pharmacy from Iasi, 16 University Street, 700115 Iasi, Romania; gabriela-dumitrita.s@umfiasi.ro (G.D.S.); bogdan.tamba@umfiasi.ro (B.-I.T.); 5 Department of Internal Medicine, Faculty of Medicine, “Grigore T. Popa” University of Medicine and Pharmacy from Iasi, 16 University Street, 700115 Iasi, Romania; bianca-stefania.profire@d.umfiasi.ro; 6 Department of Drug Industry and Pharmaceutical Biotechnology, Faculty of Pharmacy, “Grigore T. Popa” University of Medicine and Pharmacy from Iasi, 16 University Street, 700115 Iasi, Romania; alin-viorel.focsa@umfiasi.ro

**Keywords:** ibuprofen derivatives, β-cyclodextrin, release profile, analgesic assays

## Abstract

Generally, NSAIDs are weakly soluble in water and contain both hydrophilic and hydrophobic groups. One of the most widely used NSAIDs is ibuprofen, which has a poor solubility and high permeability profile. By creating dynamic, non-covalent, water-soluble inclusion complexes, cyclodextrins (CDs) can increase the dissolution rate of low aqueous solubility drugs, operating as a drug delivery vehicle, additionally contributing significantly to the chemical stability of pharmaceuticals and to reducing drug-related irritability. In order to improve the pharmacological and pharmacokinetics profile of ibuprofen, new thiazolidin-4-one derivatives of ibuprofen (**4b**, **4g**, **4k**, **4m**) were complexed with **β-CD**, using co-precipitation and freeze-drying. The new **β-CD** complexes (**β-CD-4b**, **β-CD-4g**, **β-CD-4k**, **β-CD-4m**) were characterized using scanning electronic microscopy (SEM), differential scanning calorimetry (DSC), X-ray diffraction and a phase solubility test. Using the AutoDock-VINA algorithm included in YASARA-structure software, we investigated the binding conformation of ibuprofen derivatives to **β-CD** and measured the binding energies. We also performed an in vivo biological evaluation of the ibuprofen derivatives and corresponding **β-CD** complexes, using analgesic/anti-inflammatory assays, as well as a release profile. The results support the theory that **β-CD** complexes (**β-CD-4b**, **β-CD-4g**, **β-CD-4k**, **β-CD-4m**) have a similar effect to ibuprofen derivatives (**4b**, **4g**, **4k**, **4m**). Moreover, the **β-CD** complexes demonstrated a delayed release profile, which provides valuable insights into the drug-delivery area, focused on ibuprofen derivatives.

## 1. Introduction

Cyclodextrins (CDs) are a type of cyclic oligosaccharides which contain alpha-glucopyranose units connected by alpha-1,4-linkages. The six, seven, or eight glucose units that make up the natural cyclodextrins (α, β, γ-cyclodextrin) are also found in some manufactured CDs [1,2].

Due to their bioadaptability and multi-functional properties, CDs are one of the most commonly used host macrocycles, and in recent years there has been an increased interest in using them in clinical investigations, pharmaceutical, cosmetics and personal care, food, environmental engineering, and textile industries [1,3,4].

The most widely available, least expensive, and most useful CD is **β-CD**. Due to the suitable size of its cavity, **β-CD** can form inclusion complexes with aromatic and heterocyclic molecules with different biological and pharmacological effects. The inside cavity of **β-CD** is hydrophobic while the exterior is hydrophilic, and it is filled with OH groups [2,5].

The molecular weight, chemical composition, and extremely low octanol/water partition coefficient (log *p* values) have a high impact on the CD’s permeability through biological membranes, which limits how quickly it may traverse them. The use of CDs can boost drug delivery through aqueous diffusion-controlled barriers, but it limits drug delivery through lipophilic membrane-controlled barriers [6].

By creating dynamic, non-covalent, water-soluble inclusion complexes, CDs can increase the dissolution rate of low aqueous solubility drugs, operating as a drug delivery vehicle, across the gastrointestinal tract, for example. Additionally, CDs can contribute significantly to the chemical stability of pharmaceuticals and are frequently used to reduce drug-related irritability [3,6].

Different techniques such as physical mixing, a kneading method, spray/freeze drying, co-precipitation, co-evaporation, supercritical carbon dioxide, microwave treatment, sealed-heating, milling, or a co-grinding method, are used for the preparation of inclusion complexes [1,6]. In addition, in the past two decades, researchers have introduced supercritical carbon dioxide (scCO_2_)-assisted processes as an alternative approach to overcome some limitations of the conventional methods. This method offers several benefits like precise morphology control and narrow particle size distributions while reducing or eliminating the need for organic solvents, making them environmentally friendly [7]. Various active ingredients (e.g., nimesulide, ketoprofen, simvastatin) were coprecipitated with different polymeric carriers using a supercritical antisolvent method in order to obtain polymeric drug delivery systems with an improved drug release profile [7,8].

An important part of non-steroidal anti-inflammatory drugs (NSAIDs) is non-selective cyclooxygenases (COX-1, COX-2) inhibitors that decrease the production of prostaglandins, which are linked to gastrointestinal cytoprotection, inflammation, angiogenesis, renal hemodynamics and cartilage turnover [9,10]. Based on this mechanism of action, several side effects, including acute or chronic renal failure, proteinuria, impaired cerebral perfusion, necrotizing enterocolitis and gastrointestinal bleeding, may be associated with NSAIDs treatment [11].

In order to prevent medication abuse, the US Food and Drug Administration (FDA) and the European Medicines Agency (EMA) advise that NSAIDs be provided with the lowest effective dose and for the shortest time possible [12].

Ibuprofen is one of the most widely used NSAIDs. It has been extensively researched for both safety and efficacy and is used to treat rheumatic arthritis, osteoarthritis, back pain, muscular pain, the common cold, toothache, menstrual cramps, etc. [5,13].

Generally, NSAIDs are weakly soluble in water and contain both hydrophilic and hydrophobic groups [4]. In the biopharmaceutical categorization system (BCS), ibuprofen is classified as belonging to class II, which has a poor solubility and high permeability profile [13]. There are some reports on ibuprofen complexes with **β-CD** and its derivatives. These **β-CD** complexes are widely used and made to increase ibuprofen water solubility [1]. In addition, some derivatives of ibuprofen with a thiazolidin-4-one structure proved to have an improved analgesic and anti-inflammatory profile but low solubility [14].

The aim of this study was to improve the pharmacokinetic and pharmaco-toxicological profile of some new thiazolidin-4-one derivatives of ibuprofen, synthesized by our research group, through complexation with **β-CD**. The developed inclusion complexes were physico-chemical characterized and in vivo biological evaluated, including analgesic/anti-inflammatory tests and drug release profiles.

## 2. Materials and Methods

### 2.1. Solid ***β-CD*** Complexes Preparation

#### 2.1.1. Materials

**Figure 1:**
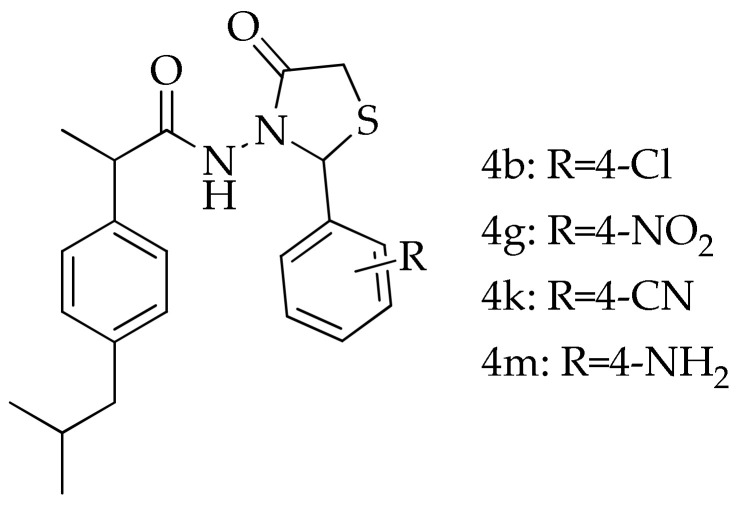
Thiazolidin-4-one derivatives of ibuprofen used for complexation with **β-CD**.

All reagents and solvents (formic acid, methanol, acetic acid, formalin, hydrochloric acid, potassium hydroxide, tween 80) were purchased from Sigma-Aldrich (Merck KGaA, Darmstadt, Germany) and were used as received. Thiazolidin-4-one derivatives of ibuprofen (**4b**, **4g**, **4k**, **4m**; Figure 1), used for complexation with **β-CD**, were synthesized by our research group, and their synthesis and spectral data were reported in a previous article [14].

#### 2.1.2. Synthesis of **β-CD** Complexes

Co-precipitation followed by freeze-drying was used to obtain the **β-CD** complexes, using a molar ratio between ibuprofen derivatives and **β-CD** of 1:1. In an aqueous solution of **β-CD** (50 mg/mL), the compound (**4b**, **4g**, **4k**, **4m**) was added and stirred continuously at room temperature for 48 h, until the solution became a suspension. The obtained mixture was introduced in liquid nitrogen and was placed for freeze-drying for 24 h in a Martin Christ, ALFA 1-2LD freeze-dryer (Osterode, Germany) [15]. The complexes were used without purification for in vivo tests.

### 2.2. Physico-Chemical Characterization of the ***β-CD*** Complexes

#### 2.2.1. Scanning Electronic Microscopy and Energy Dispersive X-ray Analysis

The elemental composition and the morphology of the obtained complexes were analyzed with a Verios G4 UC scanning electron microscope (SEM) (Thermo Scientific, Brno, Czech Republic) equipped with an energy dispersive X-ray spectroscopy analyzer (EDAX) (Octane Elect Super SDD detector, Mahwah NJ, USA). In order to provide electrical conductivity and prevent charge accumulation during exposure to the electron beam, the samples were attached to aluminum stubs with double-adhesive carbon tape and then coated with 6 nm platinum [16], using a Leica EM ACE200 Sputter coater (Vienna, Austria). An angular backscattered detector (ABS) was used in experiments, which were conducted at a 10 kV accelerating voltage.

#### 2.2.2. Differential Scanning Calorimetry

The differential scanning calorimetry (DSC) curves were registered on a DSC 200 F3 Maia device (Netzsch, Selb, Germany) calibrated with standard indium. A sample mass of 4 mg was heated in aluminum crucibles sealed shut with pierced lids. A heating rate of 10 °C/min was applied and a nitrogen atmosphere was used (flow rate of 50 mL per min).

#### 2.2.3. X-ray Diffraction

X-ray diffraction analysis was performed on a Rigaku Miniflex 600 diffractometer using CuKα emission in the angular range 3–50° (2θ) with a scanning step of 0.0025° and a recording rate of 1°/min.

#### 2.2.4. Phase Solubility Studies

Stock solutions of **β-CD** were prepared in the concentration range of 0–15 mmol/L. From each stock solution 1 mL was taken and introduced into thermomixer flasks and 1 mL of suprasaturated solution of **4b**, **4g**, **4k**, **4m** was added and stirred at 600 rpm at 25 °C for 24 h, then at 60 °C for 24 h, and finally left to rest, at 25 °C, for another 24 h. UV absorbance was monitored within 190–320 nm on extracts freshly diluted with deionized water, and the maximum wavelength of 226 nm was selected for solubility determinations. All measurements were made in triplicate.

### 2.3. Molecular Docking Studies

Molecular docking is an “in silico” method of calculating the binding energy between a ligand and a receptor. The AutoDock-VINA algorithm [17] included in YASARA-Structure software [18] was used for the calculation of the binding energy. The docking computations were performed using the AMBER force field by running a number of 400 docking trials for each ibuprofen derivative, where the receptor was considered the **β-CD**. Subsequently, cluster analysis was performed using a root-mean-square deviation (RMSD) tolerance of 5.0 Å.

### 2.4. In Vivo Biological Studies

#### 2.4.1. Animals

Swiss albino mice and Wistar rats were used and were purchased from the Cantacuzino Institute, Bucharest, Romania. Animals were housed in the animal facility of the Advanced Research and Development Center for Experimental Medicine ”Prof. Ostin C. Mungiu”-CEMEX, in individually ventilated cages (IVCs) and maintained in standard husbandry conditions: controlled room temperature (20 ± 4 °C), relative humidity (50 ± 5%) and a stress light–dark cycle; with ad libitum access to water and standard laboratory chow.

The experimental study was approved by the Ethical Committee of “Grigore T. Popa” University of Medicine and Pharmacy of Iasi (17755/03.09.2019). All procedures were carried out in compliance with the European Community Guidelines (Directive 2010/63/EU) and Romanian law (Low no. 43/2014) on the protection of animals used for scientific purposes. Every protocol step was followed with consideration for reducing animal pain and to reduce the number of animals needed for statistical significance as much as possible. At the end of the study, all animals were euthanized in accordance with the legislation.

#### 2.4.2. Analgesic Tests

The tests were performed using Swiss albino mice (25–30 g) which were divided in groups of eight mice each. The ibuprofen derivatives (**4b**, **4g**, **4k**, **4m**) and their **β-CD** complexes (**β-CD-4b**, **β-CD-4g**, **β-CD-4k**, **β-CD-4m**), were administered in a daily equivalent dose, representing 1/20 of LD50, as a suspension in tween 80, by oral gavage (Table 1). For **β-CD** complexes, the equivalent dose was established taking into account that the molar ratio between ibuprofen derivatives and **β-CD** is 1:1. Ibuprofen sodium (Ibu-Na) and tween 80, were used as a reference drug and control, respectively.

##### Tail Flick

The tail-flick test measures the reflex latency after exposure to a heating source, in order to evaluate the spinal response to pain. The tail flick unit (37360; UgoBasile, Gemonio, Italy) directs heat and stimulates the distal part of the mouse tail. The tail-flick latency is defined as the period before the mouse removed its tail from heat stimuli. The pain response at onset and at 1, 2, 3, and 4 h after administration of drugs, was noted [19,20]. To prevent tissue lesions, the experiment cut-off time was set at 12 s. The pain inhibition (%) was calculated for each tested compound/**β-CD** complex and for each group the mean value (±S.E.) was reported [14,19].

##### Hot Plate

A hot-plate test was performed using a hot plate apparatus (DS 37; UgoBasile). The plate was set at 55 ± 0.1 °C and the response latency was noted as the interval between placing the animal on the plate and the occurrence of licking, shaking of the hind paws, or jumping off the surface. The latency of the analgesic response was measured at onset and at 1, 2, 3, and 4 h. The experiment cut-off time was set to 15 s to prevent tissue damage. For each group, the mean value (±S.E.) was reported [19,20,21].

##### Writhing Test

To determine visceral pain, a writhing test was used. After 4 h from the administration of ibuprofen derivatives/**β-CD** complexes, the acetic acid (1% water solution), in a volume of 0.1 mL/10 g b.w., was intraperitoneally injected. The mice were placed in a large glass cylinder and the number of abdominal writhes was recorded over a period of 30 min. The inhibition (%) of writhings was calculated to ascertain the analgesic effect. For each group, the mean value (±S.E.) was reported [14,19,20].

##### Paw Formalin Test

The analgesic and anti-inflammatory effects of the compounds were evaluated using formalin assay. After 4 h from the administration of ibuprofen derivatives/**β-CD** complexes, a volume of 20 μL formalin (5%) was subcutaneously injected into the plantar surface of the right hind paw. The time (s) that the mice spent licking and/or biting the injected paw was recorded. Depending on the time value, the pain can be classified as neurogenic pain (0 to 5 min), interphase pain (5 to 15 min), and inflammatory pain (15 to 30 min) [19,22,23]. For each group, the mean value (±S.E.) was reported.

#### 2.4.3. Evaluation of the Drug Release Profile

##### Experimental Design

The test was performed using Wistar rats (200–300 g), which were divided in groups of five rats each. The ibuprofen derivatives (**4b**, **4g**, **4k**, **4m**) and their **β-CD** complexes (**β-CD-4b**, **β-CD-4g**, **β-CD-4k**, **β-CD-4m**), were administered, in a single equivalent dose, representing 1/20 of LD50, as a suspension in tween 80, by oral gavage (Table 1). For **β-CD** complexes, the equivalent dose was established taking into account that the molar ratio between ibuprofen derivatives and **β-CD** is 1:1. Blood samples (200 µL) were collected on anticoagulant (potassium EDTA), from the jugular vein of each rat after 1, 2, 3, 4, 6 h, under anesthesia with isoflurane. The blood was then centrifuged at 3000× *g* in the first 30 min in order to obtain plasma [15].

##### HPLC-ESI-MS Method

To quantify the ibuprofen derivatives/**β-CD** complexes in the blood plasma of rats, a high-performance liquid chromatography method coupled with electrospray ionization mass spectrometry (HPLC–ESI–MS) was developed. An Agilent 1200 Series HPLC system with a diode array detector (DAD), coupled to an Agilent 6520 accurate-mass quadrupole time-of-flight (Q-TOF) mass spectrometer equipped with electrospray ionization (ESI) source was used. Separation of ibuprofen derivatives/**β-CD** complexes was achieved on a 150 × 4.6 mm, 5 µm BDS Hypersil C18 column (Thermo Scientific) with a mobile phase consisting of 5% MiliQ water with 0.1% formic acid (A) and 95% HPLC grade methanol with 0.1% formic acid (B) applied in an isocratic mode.

Ibuprofen derivatives/**β-CD** complexes were injected in an aliquot of 10 µL each, running at a flow rate of 0.8 mL/min. The DAD acquisition was performed in the range of 200–800 nm and the separation process was recorded at 264 nm.

After DAD, an aliquot of elute was directed to ESI/Q-TOF MS at a rate of 0.1 mL/min. The device was run at a source temperature of 325 °C and an ionization voltage of −4000 V. As a drying gas, nitrogen was used, at a flow rate of 6 L/min and as a nebulizer gas, at a pressure of 25 psi. A negative ion mode and a full scan of ions in the range of 50–800 *m*/*z* were used. The mass scale was calibrated using a standard calibration method and manufacturer-supplied reference substances. MassHunter Workstation Software Data Acquisition for 6200/6500 Series, version B.01.03, was used to register and analyze data.

The retention time and MS chromatograms of compounds detected from rats’ plasma were confirmed by the calibration curves, plotted using serial dilutions of ibuprofen derivatives (**4b**, **4g**, **4k**, **4m**).

##### Preparation of Serial Dilutions

The standard curves were made using serial dilutions of stock solution in methanol of each ibuprofen derivative as follows: **4b** (stock solution of 4 mg/mL; serial dilutions with concentrations of 2.0; 1.5; 1.0; 0.8; 0.5; 0.3 mg/mL), **4g** (stock solution of 5 mg/mL; serial dilutions with concentrations of 1.0; 0.8; 0.6; 0.5; 0.3; 0.1 mg/mL), **4k** (stock solution of 15 mg/mL; serial dilutions with concentrations of 2; 1.5; 1.0; 0.8; 0.5; 0.3 mg/mL), **4m** (stock solution of 5 mg/mL; serial dilutions with concentrations of 1.5; 1.3; 1.0; 0.7; 0.5; 0.3 mg/mL).

The solutions were filtered by a 0.22 μm filter and subjected to HPLC-ESI-MS analysis using the above conditions. The calibration curves were plotted using each compound’s peak area (Y) versus the corresponding concentration (X).

##### Blood Samples Preparation

Before analyzing, the plasma samples must be deproteinized in order to prevent clogging of the chromatographic column. The deproteinization procedure was performed according to the literature procedure, where one volume of plasma was mixed with a half volume of 10% hydrochloric acid, vortexed for 20 s, and centrifuged at 5000 rpm for 10 min. The pH of the supernatant was adjusted at 7 with 4 M potassium hydroxide, and then the sample was subjected to chromatographic analysis [15,24].

## 3. Results and Discussions

### 3.1. Physico-Chemical Characterization of ***β-CD*** Complexes

#### 3.1.1. Scanning Electronic Microscopy and Energy Dispersive X-ray Analysis

**Figure 2:**
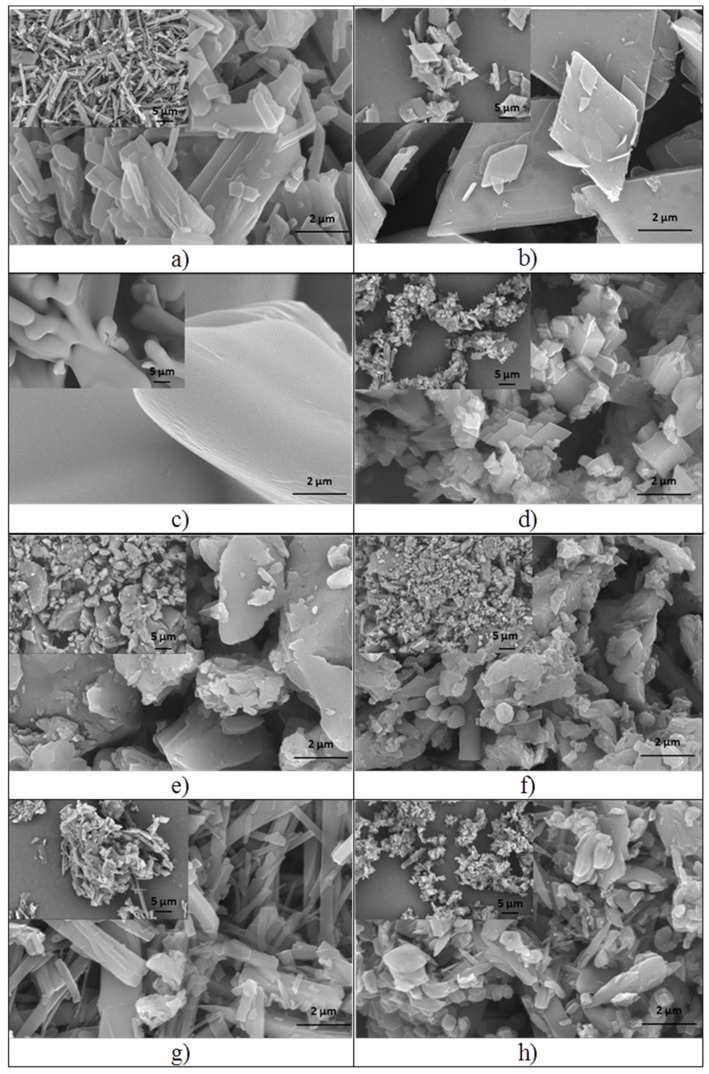
SEM morphology of ibuprofen derivatives: **4b** (**a**), **4g** (**c**), **4k** (**e**), **4m** (**g**) and their **β-CD** complexes: **β-CD-4b** (**b**), **β-CD-4g** (**d**), **β-CD-4k** (**f**), **β-CD-4m** (**h**).

The SEM micrographs recorded for the ibuprofen derivatives and their **β-CD** complexes are shown in Figure 2. The micrographs were recorded at two magnifications: 10,000X, to highlight the morphological details, and 2500X (inset), to highlight the homogeneity of the samples.

It was noted that the morphology of the ibuprofen derivatives is different from that of the corresponding **β-CD** complex, which supports the inclusion of the ibuprofen derivative into the cavity of **β-CD**. The structure of ibuprofen derivatives and the nature of the substituent of the aromatic ring (4-Cl, 4-NO_2_, 4-CN, 4-NH_2_), respectively, influence the nature of the intermolecular interactions, and so the manner in which the molecules are assembled, thus leading to structures with different morphological aspects.

Ibuprofen derivatives, **4b** and **4m**, present a crystalline structure and needle-shaped morphology, similar to ibuprofen. In the case of **4g**, a compact structure with no crystalline aggregates can be observed, while larger crystalline aggregates, with irregular shapes, can be observed in the case of **4k**. Micrographs of all **β-CD** complexes show a crystalline morphology with different shapes and sizes of particles. So, **β-CD-4b** (Figure 2b) presents three-dimensional rhomboid-shaped crystals, while **β-CD-4g** (Figure 2d) is composed of rhomboid and cubic-shaped crystals. Samples **β-CD-4k** and **β-CD-4m** exhibit crystals with irregular borders and different sizes. In conclusion, although the SEM technique cannot confirm with certainty the formation of **β-CD** complexes, based on observed morphological differences, we can conclude that there are data that indicate the formation of **β-CD** complexes.

**Figure 3:**
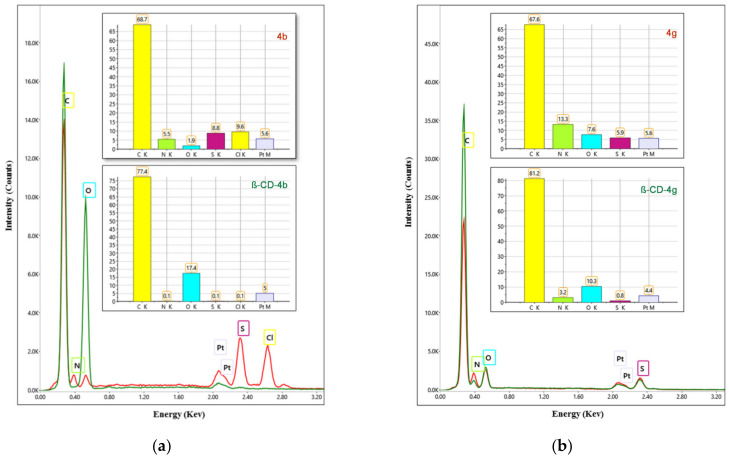

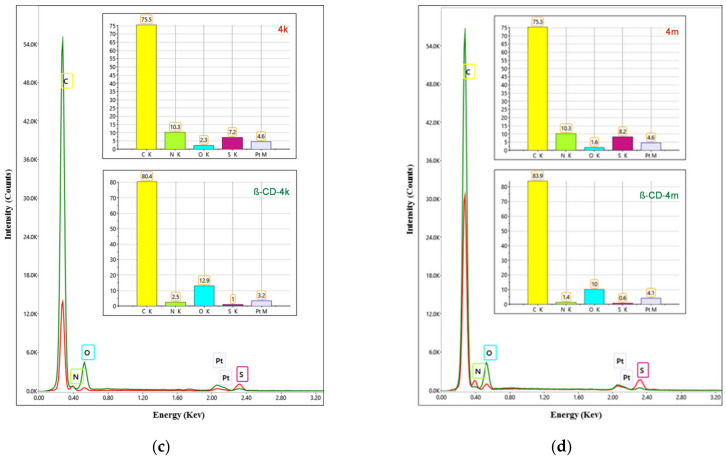
EDX spectra (red color)/**β-CD** complexes (green color) and elemental composition of ibuprofen derivative: **4b** and **β-CD-4b** (**a**), **4g** and **β-CD-4g** (**b**), **4k** and **β-CD-4k** (**c**), **4m** and **β-CD-4m** (**d**).

The EDX spectra of ibuprofen derivatives (**4b**, **4g**, **4k**, **4m**) and the corresponding **β-CD** complexes (**β-CD-4b**, **β-CD-4g**, **β-CD-4k**, **β-CD-4m**) are presented in Figure 3, which also includes the mass percentages corresponding to the identified chemical elements. EDX data for **4g**, **4k**, and **4m** revealed the presence of C, O, N, and S and for **4b**, Cl was also identified. In the case of **β-CD** complexes that contain C, O, and H (hydrogen cannot be identified by the EDX technique), the main change that can be observed is the increase in the percentage of C and O. Since the EDX analysis carried out is a qualitative one, we can say that both ibuprofen derivatives and the corresponding **β-CD** complexes do not contain other elements apart from those corresponding to their chemical structure.

#### 3.1.2. Differential Scanning Calorimetry

**Figure 4:**
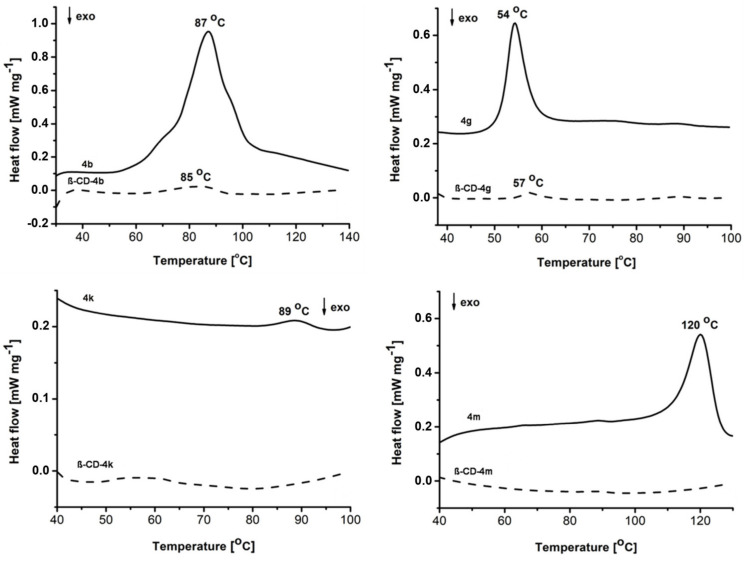
The DSC curves of the ibuprofen derivative (**4b**, **4g**, **4k**, **4m**) and **β-CD** complexes (**β-CD-4b**, **β-CD-4g**, **β-CD-4k** and **β-CD-4m**).

The formation of the solid-state host–guest interactions was evidenced by recording the DSC curves of the ibuprofen derivatives and their **β-CD** complexes (Figure 4).

According to the literature, the melting peak of pure racemic ibuprofen occurs in the range 75–85 °C [25] while for the (+) and (–) isomers it is lower (47–54 °C) [26]. This aspect is in good agreement with the melting profiles of derivatives **4b** (87 °C), **4k** (89 °C) and **4g** (54 °C). Nonetheless, the melting profile of ibuprofen derivatives is influenced by the thiazolidin-4-one moiety and, more specifically, by the nature of the R substitute. This influence is more intense for **4m**, for which the melting temperature increased at 120 °C.

In the case of **β-CD** complexes, the drug included in the cavity is thermally protected. This aspect is reflected by the decreasing or complete disappearance of the guest molecule melting profile, accompanied by the possibility of thermal displacement [27]. The analysis of the DSC curves revealed that for **β-CD-4b** and **β-CD-4g**, the melting peaks are significantly reduced and slightly displaced (85 °C and 57 °C, respectively), while for **β-CD-4k** and **β-CD-4m** the melting peaks completely disappeared (Figure 4) [28].

#### 3.1.3. X-ray Diffraction

**Figure 5:**
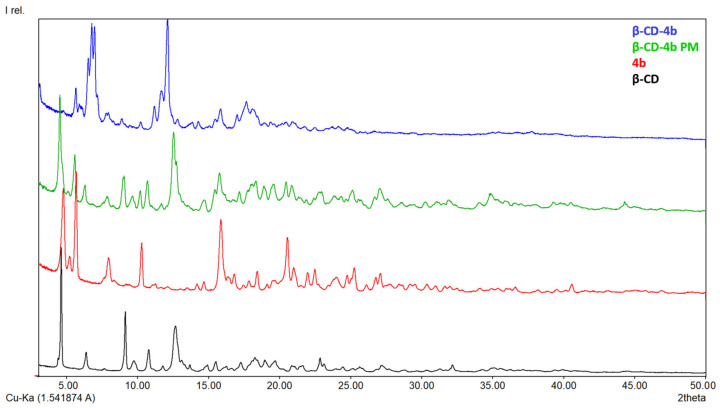
X-ray diffraction patterns of **β-CD** (black), **4b** (red), **β-CD-4b** (blue) and **β-CD-4b** PM (physical mixture, green).

**Figure 6:**
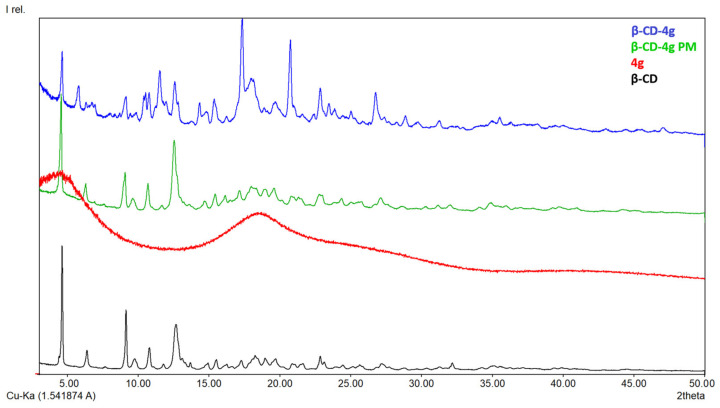
X-ray diffraction patterns of **β-CD** (black), **4g** (red), **β-CD-4g** (blue) and **β-CD-4g** PM (physical mixture, green).

**Figure 7:**
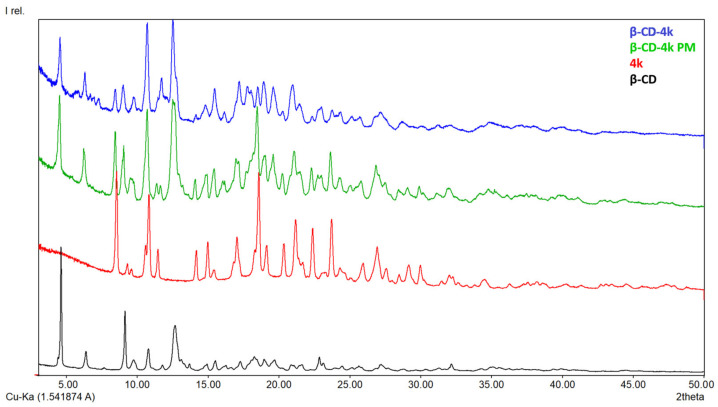
X-ray diffraction patterns of **β-CD** (black), **4k** (red), **β-CD-4k** (blue) and **β-CD-4k** PM (physical mixture, green).

**Figure 8:**
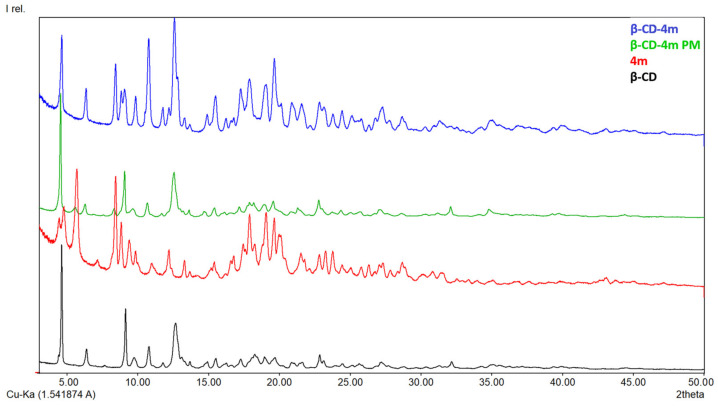
X-ray diffraction patterns of **β-CD** (black), **4m** (red), **β-CD-4m** (blue) and **β-CD-4m** PM (physical mixture, green).

In Figure 5, Figure 6, Figure 7 and Figure 8, the diffraction patterns of the pure compounds (**4b**, **4g**, **4k**, **4m**) and their physical mixtures as well as of inclusion complexes (**β-CD-4b**, **β-CD-4g**, **β-CD-4k**, **β-CD-4m**) are presented. X-ray diffractograms of physical mixtures of **4b**, **4g**, **4k**, and **4m** with **β-CD** in a 1:1 molar ratio indicate that they contain characteristic peaks of both **β-CD** and the ibuprofen derivatives. By contrast, diffractograms of inclusion complexes (**β-CD-4b**, **β-CD-4g**, **β-CD-4k**, **β-CD-4m**) present a modified diffraction pattern, with peaks wider and slightly displaced compared to those in pure compounds. In addition, the 2ϴ at around 12.7°, which is characteristic of pure **β-CD**, in the diffraction patterns of inclusion complexes are different, demonstrating a modification in the amorphization of **β-CD** or the formation of its inclusion complexes. Moreover, the presence of the peaks at 2ϴ at around 7–9° indicate the formation of channel-type structures and is characteristic of the formation of inclusion complexes or the formation of different solids with different properties [29,30].

#### 3.1.4. Phase Solubility Studies

**Figure 9:**
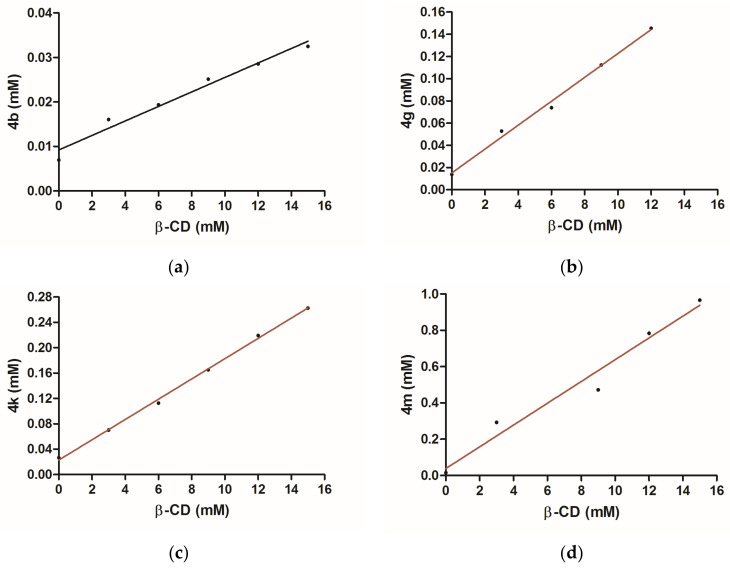
Phase solubility diagrams for **4b** (**a**); **4g** (**b**); **4k** (**c**); **4m** (**d**), correlated with **β-CD** concentration.

According to the literature, the phase solubility diagrams (Figure 9) exhibit characteristics which support their classification as an A(L)-type, showing linear increases in the solubility of ibuprofen derivatives with host concentration at room temperature within the entire solubility range of **β-CD**. Based on the observed data, it can be inferred that the inclusion complexes exhibit a 1:1 molar ratio between the ibuprofen derivatives and **β-CD**; as a result, the following equation was applied [31]:

$$K_{1:1}=\frac{Slope}{S_{0}\left(1-Slope\right)}$$

where K_1:1_ is apparent solubility constant, S_0_ is the solubility of the ibuprofen derivative in the absence of **β-CD** and the slope represents the curve’s slope of the same investigated derivative.

So values were determined from the calibration curves of each derivative in water and were found to be: 0.009209 mg/mL (**4b**), 0.01502 mg/mL (**4g**), 0.02277 mg/mL (**4k**) and 0.03811 mg/mL (**4m**).

The apparent solubility constants (K1:1) of ibuprofen derivatives in the presence of **β-CD** are: K_4b 1:1_ = 771.81 mol^−1^, K_4g 1:1_ = 723.50 mol^−1^, K_4k 1:1_ = 713.6510 mol^−1^, K_4m 1:1_ = 1 674.58 mol^−1^. As can be seen, there are some differences in the solubility constants of the studied derivatives, which means differences in their stability. This disparity is attributed to the varying capacity of distinct functional groups to engage in hydrogen bonding with **β-CD** hydroxyl groups. For instance, functional groups such as -NH_2_ (**4m**) enhance stability, while those causing electrostatic repulsion, such as –Cl (**4b**), diminish stability [32].

#### 3.1.5. Molecular Docking

**Figure 10:**
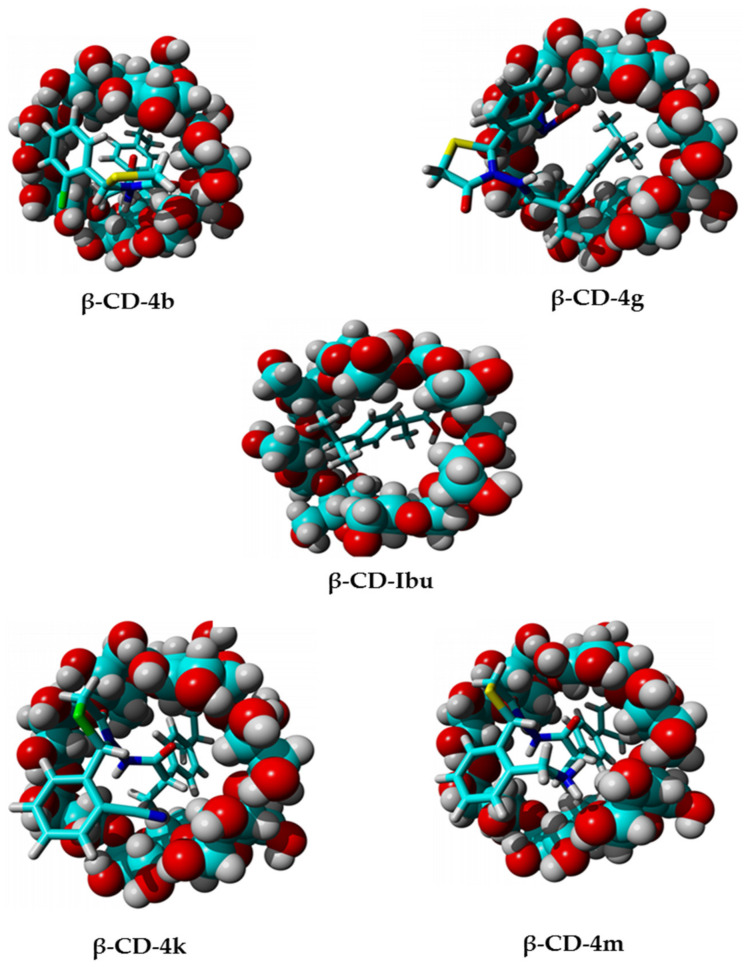
Binding conformation of ibuprofen and ibuprofen derivatives (**4b**, **4g**, **4k**, **4m**) to **β-CD**.

As can be seen in Figure 10, the ibuprofen derivatives (**4b**, **4g**, **4k**, **4m**) are entering the cavity of **β-CD**, with ibuprofen moiety firstly.

These conformations explain very well why when we use **β-CD** the water solubility is greatly improved because, as can be seen, the insoluble part of ibuprofen moiety is completely hidden inside the **β-CD** cavity. Referring to the binding energy, it can be seen that the compound **4k** forms the most stable complex, with the highest binding energy (6.305 kcal/mol), while for the un-included ibuprofen the binding energy was only 4.674 kcal/mol. The **4b** (5.785 kcal/mol), **4g** (5.838 kcal/mol), and **4m** (5.974 kcal/mol) showed similar binding energies.

### 3.2. Analgesic Tests

#### 3.2.1. Tail Flick

**Figure 11:**
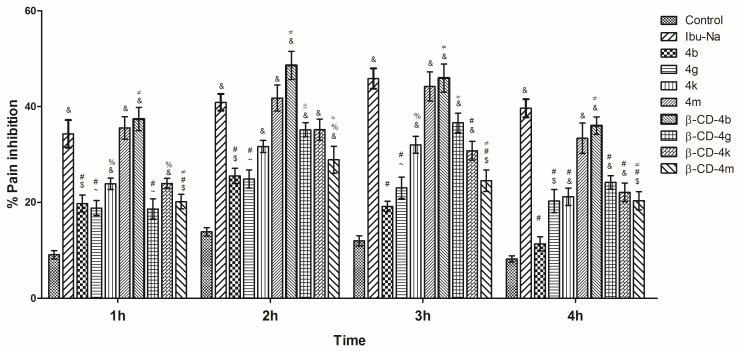
Pain inhibition (%) in tail-flick assay of ibuprofen derivatives (**4b**, **4g**, **4k**, **4m**) and their corresponding **β-CD** complexes (**β-CD-4b**, **β-CD-4g**, **β-CD-4k**, **β-CD-4m**). One-way analysis of variance (ANOVA) followed by Tukey post hoc test was performed. &, *p* < 0.001 vs. control; $, *p* < 0.01 vs. control; ~, *p* < 0.05 vs. control; #, *p* < 0.001 vs. Ibu-Na; %, *p* < 0.01 vs. Ibu-Na; ≠, *p* < 0.001 **β-CD**-complex vs. ibuprofen derivative; ≈, *p* < 0.01 **β-CD**-complex vs. ibuprofen derivative; ±, *p* < 0.05 β-CD-complex vs. ibuprofen derivative.

The results, expressed as pain inhibition percentages (%) for ibuprofen derivatives (**4b**, **4g**, **4k**, **4m**), **β-CD** complexes (**β-CD-4b**, **β-CD-4g**, **β-CD-4k**, **β-CD-4m**), in reference tothe Ibu-Na and control, at different times (1 h, 2 h, 3 h, 4 h), are presented in Figure 11. It was noted that the effect showed by **4m** and **β-CD-4b** was similar to Ibu-Na, during the whole experiment. The maximum effect was achieved at 3 h for **4m** (44.19%), **4k** (32.01%), and **β-CD-4g** (36.61%), being comparable with that of Ibu-Na (45.86%). For **4b** (25.54%), **4g** (24.90%), **β-CD-4b** (48.60%), **β-CD-4k** (35.20%), **β-CD-4m** (28.89%) the maximum effect was recorded after 2 h. It can be observed also that for **β-CD-4b**, the effect was even a little bit more intense than Ibu-Na.

#### 3.2.2. Hot Plate

**Figure 12:**
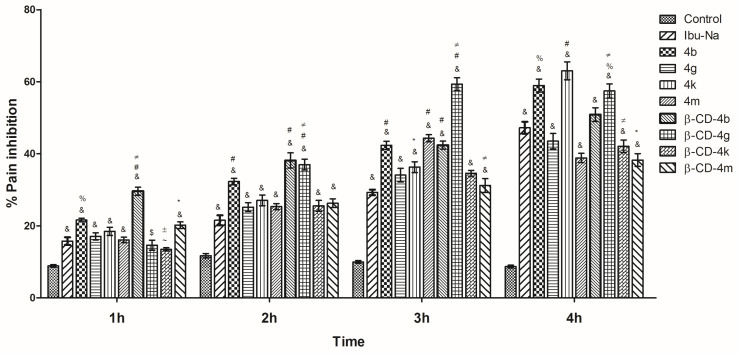
Pain inhibition (%) in hot plate assay of ibuprofen derivatives (**4b**, **4g**, **4k**, **4m**) and their corresponding **β-CD** complexes (**β-CD-4b**, **β-CD-4g**, **β-CD-4k**, **β-CD-4m**). One-way analysis of variance (ANOVA) followed by Tukey post hoc test was performed. &, *p* < 0.001 vs. control; $, *p* < 0.01 vs. control; ~, *p* < 0.05 vs. control; #, *p* < 0.001 vs. Ibu-Na; %, *p* < 0.01 vs. Ibu-Na; *, *p* < 0.05 vs. Ibu-Na; ≠, *p* < 0.001 **β-CD**-complex vs. ibuprofen derivative, ±, *p* < 0.05 **β-CD**-complex vs. ibuprofen derivative.

As can be observed (Figure 12), for most ibuprofen derivatives/**β-CD** complexes the maximum analgesic effect was achieved after 4 h, except for **4m** and **β-CD-4g** for which the maximum effect was recorded after 3 h.

It was also noted that the effect increased with time, the effect recorded after 3 h and 4 h, respectively, being more intense than those recorded at the previous time. The most intense effect was recorded for **4b**, **4k**, and **β-CD-4g**, for which, after 4 h, the pain inhibition was 58.93%, 63.03%, and 57.43%, respectively, being more active than Ibu-Na (47.22%).

#### 3.2.3. Writhing Test

**Figure 13:**
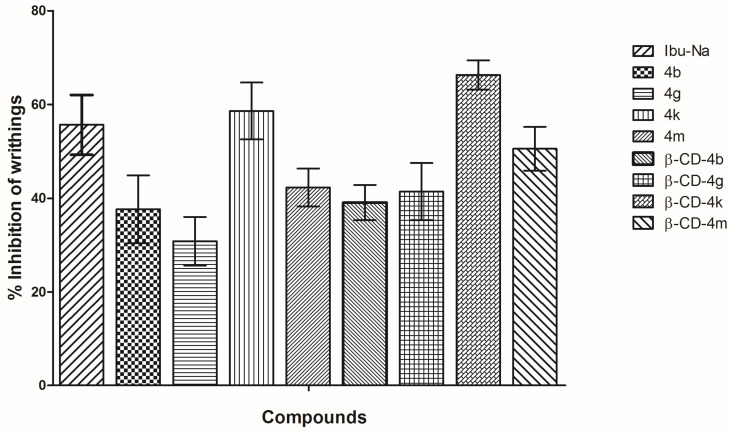
Writhings inhibition (%) of ibuprofen derivatives (**4b**, **4g**, **4k**, **4m**) and their corresponding **β-CD** complexes (**β-CD-4b**, **β-CD-4g**, **β-CD-4k, β-CD-4m**). One-way analysis of variance (ANOVA) followed by Tukey post hoc test was performed (*p* > 0.05, not statistically significant).

The writhing test is a model used to assess visceral pain inhibition by measuring abdominal contortions. The analysis of the results (Figure 13) revealed that the inhibition of writhings (%) ranged from 37.63% to 66.31%, the effect of **β-CD** complexes (**β-CD-4b**, **β-CD-4g**, **β-CD-4k**, **β-CD-4m**) being more intense than that of the corresponding ibuprofen derivatives (**4b**, **4g**, **4k**, **4m**). Among the ibuprofen derivatives, the most active was 4k (58.60%), which showed a similar effect to Ibu-Na (55.65%). A similar effect to Ibu-Na was evidenced also for **β-CD-4m** (50.53%), while for **β-CD-4k** (66.31%) the effect was higher than Ibu-Na.

#### 3.2.4. Paw Formalin Test

**Figure 14:**
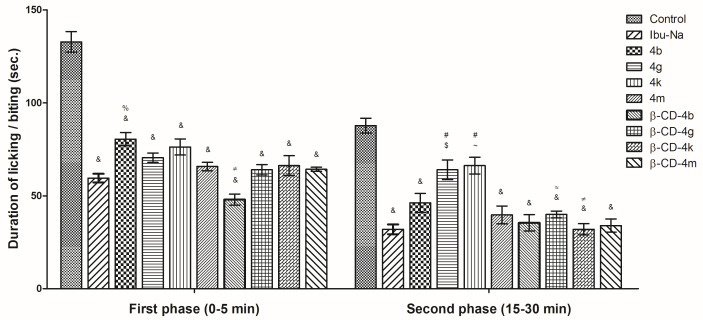
Time latencies in the paw formalin test. One-way analysis of variance (ANOVA) followed by the Tukey post hoc test was performed. &, *p* < 0.001 vs. control; $, *p* < 0.01 vs. control; ~, *p* < 0.05 vs. control; #, *p* < 0.001 vs. Ibu-Na; %, *p* < 0.01 vs. Ibu-Na; ≠, *p* < 0.001 **β-CD**-complex vs. ibuprofen derivative; ≈, *p* < 0.01 **β-CD**-complex vs. ibuprofen derivative.

The time mice spent licking their formalin-injected paws was decreased by pre-treatment with ibuprofen derivatives (**4b**, **4g**, **4k**, **4m**) and their corresponding **β-CD** complexes (**β-CD-4b**, **β-CD-4g**, **β-CD-4k**, **β-CD-4m**) (Figure 14). In the first phase (0–5 min) attributed to neurogenic pain, it can be observed that the ibuprofen derivatives and the **β-CD** complexes reduced the pain, the effect of **β-CD** complexes being more intense than that of corresponding ibuprofen derivatives. The most relevant was **β-CD**-**4m**, for which the time that mice spent licking was lower (48 s), compared with Ibu-Na (59.50 s).

For the second phase (15–30 min), correlated to inflammatory pain, all **β-CD** complexes showed similar anti-inflammatory effects with Ibu-Na (32 s) and performed better than their corresponding ibuprofen derivatives.

### 3.3. Drug Release Profile

**Figure 15:**
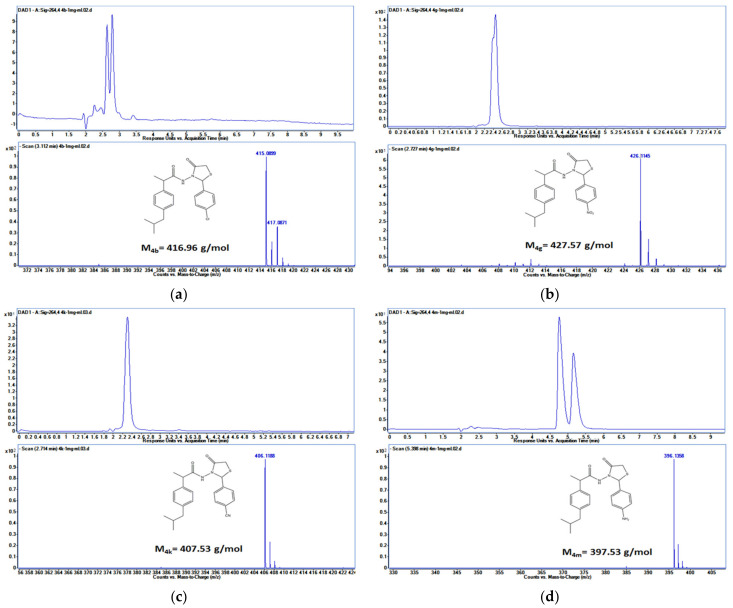
HPLC chromatogram and ESI-MS spectra recorded in negative mode for ibuprofen derivatives: **4b** (**a**); **4g** (**b**); **4k** (**c**); **4m** (**d**).

The HPLC chromatogram and ESI-MS spectra (Figure 15) proved the structure of ibuprofen derivatives (**4b**, **4g**, **4k**, **4m**), based on *m*/*z* mass data.

**Figure 16:**
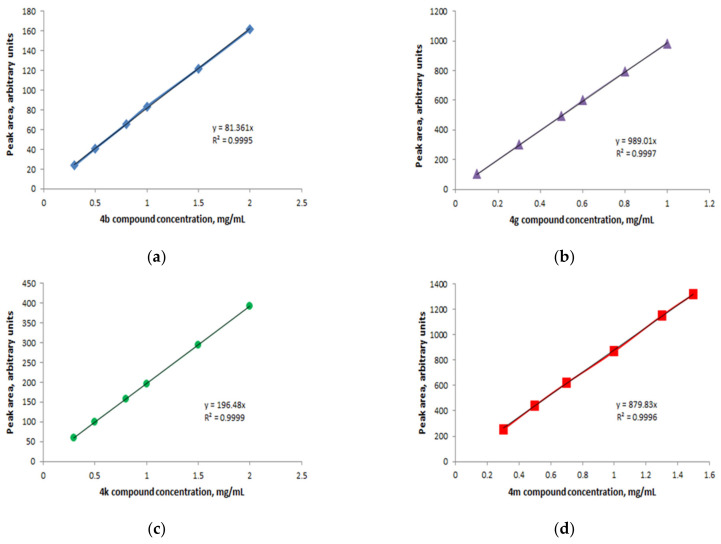
Calibration curve for ibuprofen derivatives: **4b** (**a**); **4g** (**b**); **4k** (**c**); **4m** (**d**).

Using different concentrations of ibuprofen derivatives (**4b**, **4g**, **4k**, **4m**), we plotted the calibration curve of each derivative (Figure 16) in order to quantify the concentration of ibuprofen derivatives (**4b**, **4g**, **4k**, **4m**) and their corresponding **β-CD** complexes (**β-CD-4b**, **β-CD-4g**, **β-CD-4k**, **β-CD-4m**) in rat plasma.

**Figure 17:**
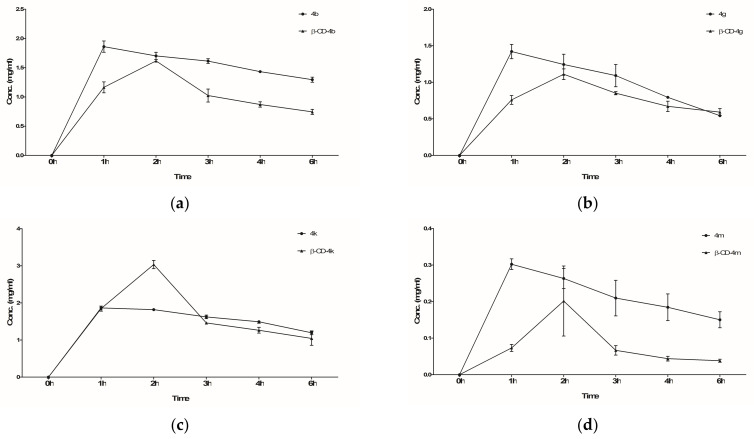
Concentration of ibuprofen derivatives (**4b**, **4g**, **4k**, **4m**) and their corresponding **β-CD** complexes (**β-CD-4b**, **β-CD-4g**, **β-CD-4k**, **β-CD-4m**) in rat plasma, at different times: **4b** and **β-CD-4b** (**a**); **4g** and **β-CD-4g** (**b**); **4k** and **β-CD-4k** (**c**); **4m** and **β-CD-4m** (**d**).

As can be seen in Figure 17, all the compounds (ibuprofen derivatives and **β-CD** complexes) were identified in rat plasma. Although the release profile was quite similar for both compounds, it could be observed that the maximum release peak was recorded after 2 h for **β-CD** complexes compared to 1h for ibuprofen derivatives. This finding supports the theory that the **β-CD** complexes exhibit a delayed release profile compared to ibuprofen derivatives, which can be explained by the inclusion of ibuprofen derivatives inside the **β-CD**s cavity. The delayed release profile could have potential implications for prolonging the therapeutic effects or enhancing targeted delivery of the ibuprofen derivatives, which could serve as a new starting point for future research.

## 4. Conclusions

In clusion complexes provide a versatile tool for improving the properties and applications of many drugs, by increasing the solubility and stability, targeted delivery, and protection of active substances. Their applications are important for a range of industries, such as pharmaceuticals, materials science, and the chemical industry. This study is focused on improving the solubility of new ibuprofen derivatives with thiazolidin-4-one scaffold (**4b**, **4g**, **4k**, **4m**) through complexation with **β-CD**, using co-precipitation and freeze-drying. The formation of **β-CD** complexes (**β-CD-4b**, **β-CD-4g**, **β-CD-4k**, **β-CD-4m**) was proved using SEM, X-ray, DSC, and a phase solubility test. The inclusion behavior, in terms of binding conformation of ibuprofen derivatives to **β-CD**, was also investigated using molecular docking. Based on the binding energy value, the most stable complex was **β-CD-4k**. Similar to ibuprofen derivatives, the corresponding **β-CD** complexes showed important analgesic and anti-inflammatory effects. Moreover, the in vivo study evidenced that the **β-CD** complexes presented the maximum concentration in the blood at 2 h in comparison with ibuprofen derivatives that showed it at 1h. This finding suggests that ibuprofen derivatives **β-CD** complexes have a delayed release profile, providing valuable insights into the drug-delivery area and can contribute to the development of more effective pharmaceutical products.

## Figures and Tables

**Table 1:** Doses of ibuprofen derivatives and their **β-CD** complexes used for analgesic and release profile tests.

Compound/Complexes	Dose/Equivalent Dose (mg/kg b.w.)
**4b/β-CD-4b**	81.25/302.41
**4g/β-CD-4g**	91.00/332.55
**4k/β-CD-4k**	78.25/296.17
**4m/β-CD-4m**	82.50/318.04
**Ibu-Na**	68.75

## Data Availability

The data could be requested from authors.
